# Stieltjes constants of *L*-functions in the extended Selberg class

**DOI:** 10.1007/s11139-021-00391-1

**Published:** 2021-03-20

**Authors:** Shōta Inoue, Sumaia Saad Eddin, Ade Irma Suriajaya

**Affiliations:** 1 Graduate School of Mathematics, Nagoya University, Furo-cho, Chikusa-ku, Nagoya, 464-8602 Japan; 2 Institute of Financial Mathematics and Applied Number Theory, JKU Linz, Altenbergerstraße 69, 4040 Linz, Austria; 3 Faculty of Mathematics, Kyushu University, 744 Motooka, Nishi-ku, Fukuoka 819-0395 Japan

**Keywords:** Laurent–Stieltjes constant, *L*-function, Extended Selberg class, Upper bound, 11N37, 11Y60

## Abstract

Let *f* be an arithmetic function and let ${\mathcal {S}}^\#$ denote the extended Selberg class. We denote by ${\mathcal {L}}(s) = \sum _{n = 1}^{\infty }\frac{f(n)}{n^s}$ the Dirichlet series attached to *f*. The Laurent–Stieltjes constants of ${\mathcal {L}}(s)$, which belongs to ${\mathcal {S}}^\#$, are the coefficients of the Laurent expansion of ${\mathcal {L}}$ at its pole $s=1$. In this paper, we give an upper bound of these constants, which is a generalization of many known results.

## Introduction

Let *q* be any positive integer $\ge 1$ and let $\chi$ be a Dirichlet character modulo *q*. Let $\gamma _n(\chi )$ denote the Laurent coefficients of the *Dirichlet L-function* $L(s, \chi )$ near $s=1$. We recall that

$$\begin{aligned} \gamma _n(\chi )=\sum _{a=1}^{q}\chi (a)\gamma _n(a, q), \end{aligned}$$

where

$$\begin{aligned} \gamma _n(a, q)=\lim \limits _{M\rightarrow \infty }\sum _{1\le m \equiv a \bmod q}^{M}\frac{(\log m)^n}{m}-\frac{(\log M)^{n+1}}{q(n+1)}. \end{aligned}$$

In particular, $\gamma _0(1,1) = 0.57721 56649\cdots$ is the well-known Euler constant. The constants $\gamma _n(a, q)$ are often called the *Stieltjes constants* or *generalized Euler constants*. In the particular case when $\chi =\chi _0$, where $\chi _0$ is the principal character modulo 1, the Dirichlet *L*-function $L(s, \chi _0)$ reduces to the *Riemann zeta function* $\zeta (s)$, that is $L(s, \chi _0)=\zeta (s)$. We write the corresponding Laurent coefficients simply $\gamma _n(\chi _0)=\gamma _n(1,1)=\gamma _n$. Stieltjes in 1885 showed that

1 $$\begin{aligned} \gamma _n= \frac{(-1)^n}{n!}\lim \limits _{M\rightarrow \infty }\left( \sum \limits _{m=1}^{M}\frac{(\log m)^n }{m}-\frac{(\log M)^{n+1}}{(n+1)}\right) , \end{aligned}$$

which pioneered the study of Laurent coefficients of zeta functions and *L*-functions. This gives rise to the widely used name “Stieltjes constants” for these coefficients.

The asymptotic behavior of $\gamma _n$ as $n\rightarrow \infty$ has been widely studied by many authors (for instance: Briggs [5], Mitrovic̀ [13], Israilov [10], Matsuoka [12], and more recently, Coffey [6, 7], Knessl and Coffey [11], Adell [2], Adell and Lekuona [1], and Saad Eddin [16]). The studies mostly focused on the growth and sign changes of the sequence $(\gamma _n)$, explicit upper estimates for $|\gamma _n|$, and asymptotic expressions for $\gamma _n$. Stieltjes constants for other zeta functions and *L*-functions have also been studied by many authors. We introduce some of their results in the following section.

In this paper, we are interested in investigating the Stieltjes constants of more general *L*-functions. We consider functions in a class larger than the Selberg class. We first introduce the Selberg class ${\mathcal {S}}$.

### Selberg class

Let *f* be an arithmetic function, and denote by ${\mathcal {L}}(s) = \sum _{n = 1}^{\infty }\frac{f(n)}{n^s}$ the Dirichlet series attached to *f*. We say the Dirichlet series ${\mathcal {L}}(s)$ belongs to the Selberg class ${\mathcal {S}}$ if it is absolutely convergent when ${\text {Re}}(s)>1$ and satisfies the following properties:

#### Condition

${\varvec{{\mathcal {S}}}}\,1$ Ramanujan hypothesis: For any $\varepsilon > 0$, we have $f(n) \ll _{\varepsilon } n^{\varepsilon }$.

#### Condition

${\varvec{{\mathcal {S}}}}\,2$ Analytic continuation: There exists $k \in {\mathbb {Z}}_{\ge 0}$ such that $(s - 1)^{k}{\mathcal {L}}(s)$ is entire of finite order.

#### Condition

${\varvec{{\mathcal {S}}}}\,3$ Functional equation: Define

$$\begin{aligned} {\mathcal {F}}(s) {:}{=} Q^{s}\prod _{j = 1}^{r}\Gamma (\lambda _{j}s + \mu _{j}), \end{aligned}$$

where $Q, \lambda _i$ are positive real numbers, $\Gamma$ is the gamma function, $\mu _j$ is a complex number satisfying $\mathrm {Re}(\mu _j)\ge 0$. Then the function $\Phi (s){:}{=}{\mathcal {F}}(s){\mathcal {L}}(s)$ satisfies the functional equation

$$\begin{aligned} \Phi (s) = \omega \overline{\Phi (1 - {\overline{s}})}, \end{aligned}$$

where $\omega$ is a complex number with $|\omega | = 1$.

#### Condition

${\varvec{{\mathcal {S}}}}\,4$ Euler product: For $\mathrm {Re}(s) > 1$, the function ${\mathcal {L}}(s)$ can be written as a product over prime numbers *p*:

$$\begin{aligned} {\mathcal {L}}(s) = \prod _{p}{\mathcal {L}}_{p}(s), \end{aligned}$$

where

$$\begin{aligned} {\mathcal {L}}_{p}(s) = \exp \left( \sum _{k = 1}^{\infty }\frac{b( p^{k})}{p^{ks}} \right) , \end{aligned}$$

with $b(n) \ll n^{\theta }$, for some $\theta < \frac{1}{2}$.

All functions ${\mathcal {L}}$ in ${\mathcal {S}}$ are expected to satisfy the Riemann hypothesis. If we remove any of the above conditions, however, this is no longer true. In this particular case, the hypothesis is usually called the Grand Riemann Hypothesis: all non-trivial zeros of ${\mathcal {L}}(s)\in {\mathcal {S}}$ lie on $\mathrm {Re}(s)=1/2$. The extended Selberg class ${\mathcal {S}}^\#$ is defined to be the class of functions ${\mathcal {L}}(s) = \sum _{n = 1}^{\infty }\frac{f(n)}{n^s}$ satisfying the above conditions ${\varvec{{\mathcal {S}}}}2$and ${\varvec{{\mathcal {S}}}}3$, but not necessarily ${\varvec{{\mathcal {S}}}}1$ and ${\varvec{{\mathcal {S}}}}4$.

Notable examples of functions in ${\mathcal {S}}$ are the Riemann zeta function $\zeta (s)$, Dirichlet *L*-functions $L(s,\chi )$ associated with non-principal primitive characters $\chi$, and the Dedekind zeta function $\zeta _K(s)$ of a number field *K*. The sum of the parameters $\lambda _j$ in $\mathcal {S}$3 gives the *degree* of the *L*-function ${\mathcal {L}}(s)$ in ${\mathcal {S}}^\#$, and so in ${\mathcal {S}}$, as follows:

$$\begin{aligned} d_{\mathcal {L}} = 2 \sum _{j=1}^r \lambda _j. \end{aligned}$$

It is not known if $d_{\mathcal {L}}\in {\mathbb {Z}}_{>0}$ for all ${\mathcal {L}}\in {\mathcal {S}}$ but the degree $d_{\mathcal {L}}$ characterizes certain properties of the functions ${\mathcal {L}}\in {\mathcal {S}}$. Although the functional equation is not unique, the degree $d_{\mathcal {L}}$ is well defined and captures the growth of ${\mathcal {L}}$. We shall not discuss $d_{\mathcal {L}}$ further since it is irrelevant to the aim of this paper. The readers may refer to [19, Chap. 6] for more details about the Selberg class.

From now on we keep our focus on ${\mathcal {L}}\in {\mathcal {S}}^\#$. That is, we would like to extend beyond the Selberg class by eliminating conditions ${\varvec{{\mathcal {S}}}}1$ and ${\varvec{{\mathcal {S}}}}4$. Consider the Laurent expansion of ${\mathcal {L}}(s)$ at its possible pole $s=1$ written in the following form:

$$\begin{aligned} {\mathcal {L}}(s) = \sum _{n= -k}^{\infty }\gamma _n({\mathcal {L}})(s - 1)^{n}. \end{aligned}$$

We call the coefficients $\gamma _n({\mathcal {L}})$ the *generalized Laurent–Stieltjes constants* or the *Laurent–Stieltjes constants of the extended Selberg class*. In this paper, we study these coefficients and give an upper bound of $\gamma _n({\mathcal {L}})$ for ${\mathcal {L}}\in {\mathcal {S}}^\#$.

Our main theorem is stated as follows.

#### Theorem

Let ${\mathcal {L}}\in {\mathcal {S}}^\# \setminus \{0\}$ and let $d_{\mathcal {L}}$ be the degree of ${\mathcal {L}}$. Let *Q* be the positive real number appearing in condition ${\varvec{{\mathcal {S}}}}3$ and let

$$\begin{aligned} \lambda _{m} {:}{=} \min _{1 \le j \le r}\lambda _{j},\quad \lambda _{M} {:}{=} \max _{1 \le j \le r}\lambda _{j},\quad \mathrm {and} \quad \mu _{M} {:}{=} \max _{1 \le j \le r}|\mu _{j}|. \end{aligned}$$

For a positive integer *n* with

$$\begin{aligned} \frac{n}{\log n} > \left( \frac{1}{2} + \frac{\mu _{M} + 1}{\lambda _{m}} \right) d_{\mathcal {L}} \log (Q+3), \end{aligned}$$

we have

$$\begin{aligned}&|\gamma _n({\mathcal {L}})| \le C_{{\mathcal {L}}}(a) a^{-n} \left( 2 + \frac{1}{n - \frac{d_{\mathcal {L}}(2a - 1)}{2}} \right) , \end{aligned}$$

where *a* satisfies $1 + \frac{\mu _{M} + 1}{\lambda _{m}}< a < \frac{1}{2} + \frac{n}{d_{\mathcal {L}}}$ and

$$\begin{aligned} C_{{\mathcal {L}}}(a)&= \frac{2^rQ^{2a - 1}}{\pi } \exp \left( \frac{1}{5}\sum _{j = 1}^{r} \frac{1}{\lambda _{j}(a - 1) - \mathrm {Re}(\mu _{j})} \right) \\&\quad \times \, \left( \sum _{m = 1}^{\infty }\frac{|f(m)|}{m^{a}}\right) (8\lambda _{M}^2a^2)^{\frac{d_{\mathcal {L}}}{4}(2a - 1)}. \end{aligned}$$

Finally we remark that the Laurent–Stieltjes constants of zeta and *L*-functions have many applications not only in analytic number theory, but also in algebraic number theory and even fields outside of number theory. They can be used to determine zero-free regions for $L(s,\chi )$ near the real axis in the critical strip $0\le \mathrm {Re}(s)\le 1$, to compute the values of $\zeta (s)$ in the complex plane, to study the class number of a quadratic field, etc. Further, they appear in an explicit expression of Li’s criterion which relates them to the Riemann hypothesis, see [4].

## Some known results on the Laurent–Stieltjes constants of zeta and *L*-functions

The first explicit upper bound for $|\gamma _n|$ has been given by Briggs [5], which was later improved by Berndt [3] and Israilov [10]. In 1985, the theory made a huge progress via an asymptotic expansion shown by Matsuoka [12], for these constants. He gave an excellent upper bound of $|\gamma _n|$ for $n\ge 10$ and proved that

$$\begin{aligned} |\gamma _n|\le 10^{-4} e^{n\log \log n}. \end{aligned}$$

This result had been the best upper bound of $|\gamma _n|$ for more than 20 years. Thanks to the above result, Matsuoka showed that $\zeta (s)$ has no zeros in the region $|s-1|\le \sqrt{2},$ with $0\le \mathrm {Re}{(s)}\le 1$. Many have tried to improve on the Matsuoka bound, with few successful attempts. Matsuoka’s work relied on a formula that is essentially a consequence of Cauchy’s integral theorem and the functional equation. More recently, the second author [16] extended this formula to Dirichlet *L*-functions. She gave the following upper bound for $|\gamma _n(\chi )|$ for primitive Dirichlet characters $\chi$ modulo *q* and for every $1\le q \le \pi e^{(n+1)/2}/(2n+2)$. We have

$$\begin{aligned} \frac{|\gamma _n(\chi )|}{n!}\le q^{-1/2}C(n, q)\min \left( 1+D(n,q), \frac{\pi ^2}{6}\right) , \end{aligned}$$

where

$$\begin{aligned} C(n, q)= 2\sqrt{2}\exp \left\{ -(n+1)\log \theta (n, q)+\theta (n, q)\left( \log \theta (n, q)+\log \frac{2q}{\pi e}\right) \right\} \end{aligned}$$

and

$$\begin{aligned} \theta (n,q)=\frac{n+1}{\log \frac{2q(n+1)}{\pi }}-1, \quad D(n, q)=2^{-\theta (n, q)-1}\frac{\theta (n, q)+1}{\theta (n, q)-1}. \end{aligned}$$

In the case when $\chi =\chi _0$ and $q=1$, this leads to a sizable improvement of Matsuoka’s bound and of previous results. As an application of this upper bound, the second author showed in [17] that this result enables us to approximate $L(s, \chi )$ in the neighborhood of $s=1$ by a short Taylor polynomial. For $N=4\log q$ and $q\ge 150$, we have

$$\begin{aligned} \left| L(s, \chi )-\sum _{n\le N}\frac{(-1)^n\gamma _n(\chi )}{n!}(s-1)^n\right| \le \frac{32.3}{q^{2.5}}, \end{aligned}$$

where $|s-1|\le e^{-1}$. She also proved that the function $\zeta (s)$ has no zeros in the region $|s-1|\le 2.2093$ with $0\le \mathrm {Re}{(s)}\le 1.$ This result is an improvement of Matsuoka’s result.

Finally, let *K* be a number field and ${\mathcal {O}}_K$ be its ring of integers. Define for $\mathrm {Re}(s)>1$ the *Dedekind zeta function*

$$\begin{aligned} \zeta _K(s)=\sum _{{\mathfrak {a}}} \frac{1}{{N{\mathfrak {a}}}^{s}} = \prod _{{\mathfrak {p}}}\frac{1}{1-{N{\mathfrak {p}}}^{-s}}, \end{aligned}$$

where ${\mathfrak {a}}$ runs over non-zero ideals in ${{\mathcal {O}}}_K$, ${\mathfrak {p}}$ runs over the prime ideals in ${{\mathcal {O}}}_K$ and ${N{\mathfrak {a}}}$ is the norm of ${\mathfrak {a}}$. It is known that $\zeta _K(s)$ can be analytically continued to ${\mathbb {C}}\setminus \{1\}$, and that at $s=1$ it has a simple pole, with residue $\gamma _{-1}(K)$ given by the analytic class number formula:

$$\begin{aligned} \gamma _{-1}(K)= \frac{2^{r_1}(2\pi )^{r_2}h(K)R(K)}{\omega (K)\sqrt{|D(K)|}}. \end{aligned}$$

Here we denote by $r_1$ the number of real embeddings of *K*, $r_2$ the number of complex embeddings of *K*, *h*(*K*) the class number of *K*, *R*(*K*) the regulator of *K*, $\omega (K)$ the number of roots of unity contained in *K* and *D*(*K*) the discriminant of the extension $K/{\mathbb {Q}}$. Consider the Laurent expansion

$$\begin{aligned} \zeta _K(s)= \frac{\gamma _{-1}(K)}{s-1} + \sum _{n=0}^{\infty }\gamma _n(K)(s-1)^n \end{aligned}$$

of $\zeta _K(s)$ at $s=1$. The constants $\gamma _n(K)$ are sometimes called the Stieltjes constants associated with the Dedekind zeta function. In [8] they are called higher Euler’s constants of *K*. The second author [18] studied these constants and showed that, for $n\ge 1$, we have

$$\begin{aligned} \gamma _n(K)= \frac{(-1)^n}{n!}\lim _{x \rightarrow \infty } \left( \sum _{N{{\mathfrak {a}}}\le x} \frac{(\log N{{\mathfrak {a}})^n}}{N{{\mathfrak {a}}}}-\gamma _{-1}(K)\frac{(\log x)^{n+1}}{n+1}\right) , \end{aligned}$$

and

$$\begin{aligned} \gamma _0(K)=\lim _{x \rightarrow \infty } \left( \sum _{N{{\mathfrak {a}}}\le x} \frac{1}{N{{\mathfrak {a}}}}-\gamma _{-1}(K)\log x\right) +\gamma _{-1}(K). \end{aligned}$$

To conclude this section, we remark that only the first constant $\gamma _K=\gamma _0(K)/\gamma _{-1}(K)$, called the Euler–Kronecker constant, which is closely related to values of the logarithmic derivative of *L*-functions, has been studied so far. For more details see for example [9, 14, 20]. This raises questions on the other Stieltjes constants associated with $\zeta _K(s)$. The authors were motivated to give partial answers to these questions in a much more general context, that is, for all *L*-functions in the extended Selberg class.

## Auxiliary lemmas

In order to prove our main result, we first show a proposition and two necessary lemmas. Recall the notation used when we defined ${\mathcal {S}}^\#$ in Sect. 1.

### Lemma 1

Let ${\mathcal {L}}\in {\mathcal {S}}^\#$ and let $d_{\mathcal {L}}$ be the degree of ${\mathcal {L}}$. Then we have

2 $$\begin{aligned} {\mathcal {L}}(\sigma + it) \asymp _{{\mathcal {L}}} |t|^{d_{\mathcal {L}}(\frac{1}{2} - \sigma )}|{\mathcal {L}}(1 - \sigma + it)| \end{aligned}$$

as $|t|\rightarrow \infty$. In particular,

3 $$\begin{aligned} {\mathcal {L}}(\sigma + it) \ll _{{\mathcal {L}}, \varepsilon } {\left\{ \begin{array}{ll} |t|^{\varepsilon } &{}\text {if }\; \sigma \ge 1, \\ |t|^{\frac{1 - \sigma }{2}d_{\mathcal {L}} + \varepsilon } &{}\text {if }\; 0 \le \sigma \le 1, \\ |t|^{(\frac{1}{2} - \sigma )d_{\mathcal {L}} + \varepsilon } &{}\text {if }\; \sigma \le 0. \end{array}\right. } \end{aligned}$$

### Proof

For the standard case when we assume $\mathcal {S}$1, see [19, Theorem 6.8]. Note here that the first-half (2) is obtained from the functional equation $\mathcal {S}$3.

Now without $\mathcal {S}$1, we note that the absolute convergence of the Dirichlet series and partial summation imply for any $\varepsilon > 0$,

4 $$\begin{aligned} \sum _{n\le x} |f(n)| \ll _{\varepsilon } x^{1+\varepsilon }. \end{aligned}$$

Note that this is weaker than $\mathcal {S}$1, but is sufficient for our purpose. Using (4) in combination with the functional equation $\mathcal {S}$3, we can easily show the bounds (3) for the case $\sigma \ge 1 + \varepsilon$ and $\sigma \le -\varepsilon$. More precisely,

$$\begin{aligned} {\mathcal {L}}(\sigma + it) \ll _{{\mathcal {L}}, \varepsilon } {\left\{ \begin{array}{ll} 1 &{}\text {if } \sigma \ge 1 + \varepsilon , \\ |t|^{(\frac{1}{2} - \sigma )d_{\mathcal {L}} + \varepsilon } &{}\text {if } \sigma \le -\varepsilon . \end{array}\right. } \end{aligned}$$

Since the function ${\mathcal {L}}(s)$ is entire of finite order from condition ${\varvec{{\mathcal {S}}}}2$, for any $\delta > 0$,

$$\begin{aligned} {\mathcal {L}}(\sigma + it) \ll _{{\mathcal {L}}, \varepsilon } \exp \exp (\delta |t|) \end{aligned}$$

holds in the strip $-1 \le \sigma \le 2$. Substituting this into (2), we can show that this also holds for $0 \le \sigma \le 1/2$. Applying the theorem of Phragmén–Lindelöf [15, Proposition 8.15], we have

$$\begin{aligned} {\mathcal {L}}(\sigma + it) \ll _{{\mathcal {L}}, \varepsilon } |t|^{\frac{1 - \sigma }{2}d_{\mathcal {L}} + \varepsilon } \end{aligned}$$

for $$0 \le \sigma \le 1$$. $$\square $$

### Proposition 1

Let ${\mathcal {L}}\in {\mathcal {S}}^\#$ with degree $d_{\mathcal {L}}>0$. Let *n* be an integer with $n > \max \left\{ 0, \frac{d_{\mathcal {L}}}{2} - 1 \right\}$. For $1< a < \frac{n + 1}{d_{\mathcal {L}}} + \frac{1}{2}$ such that $\lambda _j(1-a)+\mathrm {Re}(\mu _j)\notin {\mathbb {Z}}$ for each $j=1,2,\ldots ,r$, we have

$$\begin{aligned} \gamma _n({\mathcal {L}}) = \frac{(-1)^{n}}{2 \pi i } \int _{a - i\infty }^{a + i\infty } \frac{G_{{\mathcal {L}}}(s)}{s^{n + 1}}\overline{{\mathcal {L}}({\overline{s}})}\mathrm{d}s, \end{aligned}$$

where the function $G_{\mathcal {L}}$ is defined by

5 $$\begin{aligned} G_{\mathcal {L}}(s) {:}{=} \frac{\omega Q^{2s - 1}}{\pi ^r} \prod _{j = 1}^{r}\Gamma (\lambda _{j}s + {\overline{\mu }}_{j}) \sin (\pi (\lambda _{j}(1-s) + \mu _{j})) \Gamma (\lambda _{j}(s - 1) + 1 - \mu _{j}). \end{aligned}$$

Here $Q, \lambda _i$ are positive real numbers, $\mu _j$ and $\omega$ are complex numbers with $\mathrm {Re}(\mu _j)\ge 0$ and $|\omega | = 1$.

### Proof

**Fig. 1:**
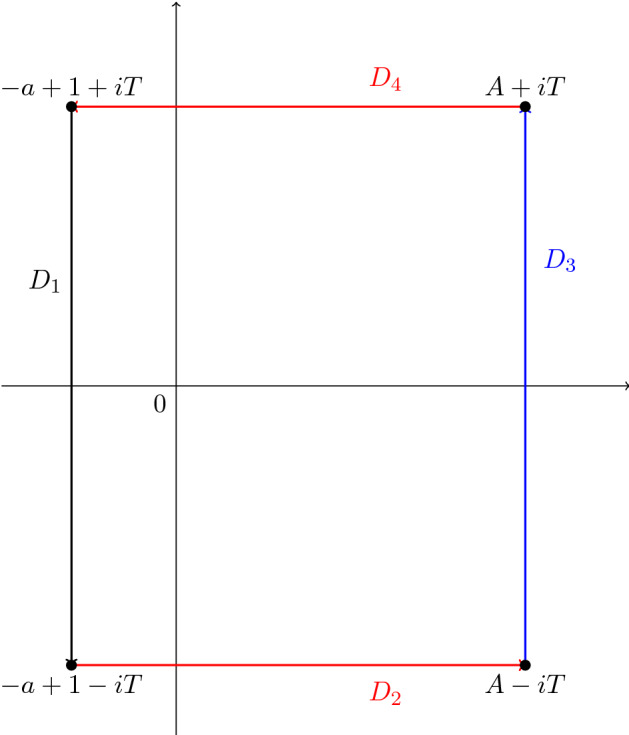
The rectangle *D* in the complex *s* plane

By Cauchy’s formula, we can write

$$\begin{aligned} \gamma _n({\mathcal {L}}) = \frac{1}{2 \pi i}\int _{D} \frac{{\mathcal {L}}(s)}{(s - 1)^{n + 1}}\mathrm{d}s, \end{aligned}$$

where *D* is the positively oriented rectangular path passing through the vertices $-a+1+iT$, $-a+1-iT$, $A-iT$ and $A+iT$, where *A* and *T* are sufficiently large numbers. Let us now divide *D* into the line segments $D_1, D_2, D_3$ and $D_4$ joining $-a+1+iT$, $-a+1-iT$, $A-iT$, $A+iT$ and $-a+1 +iT$, as in Fig. 1. Then, we have

$$\begin{aligned} \gamma _n({\mathcal {L}}) = \frac{1}{2 \pi i}\left( \int _{D_1} + \int _{D_2}+ \int _{D_3}+ \int _{D_4}\right) \frac{{\mathcal {L}}(s)}{(s - 1)^{n + 1}}\mathrm{d}s. \end{aligned}$$

By Lemma 1, the integral over $D_2$ is bounded by

$$\begin{aligned} \left| \int _{D_2}\frac{{\mathcal {L}}(s)}{(s - 1)^{n + 1}}\mathrm{d}s \right|&= \left| \left( \int _{-a + 1 - iT}^{- iT} + \int _{- iT}^{1 - iT} + \int _{1 - iT}^{A - iT}\right) \frac{{\mathcal {L}}(s)}{(s- 1)^{n + 1}}\mathrm{d}s \right| \\&\ll _{{\mathcal {L}}, \varepsilon } T^{-n-1 + \varepsilon }\\&\quad \times \,\left( \int _{-a + 1}^{0}T^{d_{\mathcal {L}}(\frac{1}{2} - \sigma )}\mathrm{d}\sigma + \int _{0}^{1}T^{\frac{1}{2}d_{\mathcal {L}}(1 - \sigma )}\mathrm{d}\sigma + \int _{1}^{A}\mathrm{d}\sigma \right) \\&\ll _{{\mathcal {L}}, \varepsilon } T^{-n - 1 + d_{\mathcal {L}}(a - 1/2) + \varepsilon }. \end{aligned}$$

Since $a < \frac{n + 1}{d_{\mathcal {L}}} + \frac{1}{2}$, the last term vanishes as $T \rightarrow +\infty$. Therefore, the integral over $D_2$ tends to 0 as $T \rightarrow +\infty$. A similar argument shows that the integral over $D_4$ tends to 0 as $T \rightarrow +\infty$.

Next we consider the integral over $D_3$. For $n > 0$, we find that

$$\begin{aligned} \left| \int _{D_3}\right| \ll _{{\mathcal {L}}, n} \int _{0}^{\infty }\frac{\mathrm{d}t}{\left( (A - 1)^2 + t^2\right) ^{(n + 1)/2}} < +\infty \end{aligned}$$

and for any $t \ge 0$,

$$\begin{aligned} \lim _{A \rightarrow + \infty }\frac{1}{\left( (A - 1)^2 + t^2 \right) ^{(n + 1)/2}} = 0. \end{aligned}$$

Hence by Lebesgue’s convergence theorem, we have $\displaystyle \lim _{A \rightarrow + \infty }\lim _{T \rightarrow +\infty }\int _{D_3} = 0$.

Thus, for $n > \max \left\{ 0, \frac{d_{\mathcal {L}}}{2} - 1 \right\}$, we have

$$\begin{aligned} \gamma _n({\mathcal {L}}) = \frac{1}{2 \pi i}\int _{-a + 1 + i\infty }^{-a + 1 - i\infty } \frac{{\mathcal {L}}(s)}{(s - 1)^{n + 1}}\mathrm{d}s = \frac{(-1)^{n}}{2\pi i}\int _{a - i\infty }^{a + i\infty }\frac{{\mathcal {L}}(1 - s)}{s^{n + 1}}\mathrm{d}s. \end{aligned}$$

Here, by using the functional equation $\mathcal {S}$3 for ${\mathcal {L}}(s)$ and the formula $\Gamma (s)\Gamma (1-s)\sin (\pi s) = \pi$, we have

$$\begin{aligned} {\mathcal {L}}(1 - s)&= \overline{{\mathcal {L}}({\overline{s}})}\left( \omega \frac{\overline{{\mathcal {F}}({\overline{s}})}}{{\mathcal {F}}(1 - s)}\right) = \overline{{\mathcal {L}}({\overline{s}})}\left( \omega Q^{2s - 1} \prod _{j = 1}^{r}\frac{\Gamma (\lambda _{j}s + \overline{\mu _{j}})}{\Gamma (\lambda _{j}(1 - s) + \mu _{j})} \right) \\&= \overline{{\mathcal {L}}({\overline{s}})}G_{\mathcal {L}}(s). \end{aligned}$$

Hence

$$\begin{aligned} \gamma _n({\mathcal {L}}) = \frac{(-1)^{n}}{2\pi i } \int _{a - i\infty }^{a + i\infty } \frac{G_{\mathcal {L}}(s)}{s^{n + 1}}\overline{{\mathcal {L}}({\overline{s}})}\mathrm{d}s, \end{aligned}$$

where the function $G_{\mathcal {L}}(s)$ is as defined in (5). This completes the proof of Proposition 1. $\square$

### Lemma 2

For ${\mathcal {L}}\in {\mathcal {S}}^\# \setminus \{0\}$, consider $G_{\mathcal {L}}$ as defined in Proposition 1. Let $\lambda _m{:}{=} \min _{1\le j \le r} \lambda _j$ and $\mu _M{:}{=} \max _{1\le j \le r} |\mu _j|$. For $a > 1 + \frac{\mu _{M}}{\lambda _{m}}$ , we have

$$\begin{aligned} |G_{\mathcal {L}}(a + it)| \le c_{{\mathcal {L}}}(a)Q^{2a - 1}\left( (a\lambda _{M} + \mu _{M} + 1)^2 + (\lambda _{M}|t| + \mu _{M})^2 \right) ^{\frac{d_{\mathcal {L}}}{4}(2a - 1)}, \end{aligned}$$

where the constant $c_{{\mathcal {L}}}(a)$ is defined by

$$\begin{aligned} c_{{\mathcal {L}}}(a) = 2^{r}\exp \left( \frac{1}{5}\sum _{j = 1}^{r} \frac{1}{\lambda _{j}(a - 1) - \mathrm {Re}(\mu _{j})} \right) . \end{aligned}$$

### Proof

Put $\lambda _m{:}{=} \min _{1\le j \le r} \lambda _j$, $\lambda _M{:}{=} \max _{1\le j \le r} \lambda _j$, $\mu _M{:}{=} \max _{1\le j \le r} |\mu _j|$, and let $a > 1 + \frac{\mu _{M}}{\lambda _{m}}$. From (5), we have

6 $$\begin{aligned} \begin{aligned} |G_{\mathcal {L}}(a + it)|&\le \frac{Q^{2a - 1}}{\pi ^{r}} \prod _{j = 1}^{r} \left| \Gamma (\lambda _{j}(a + it) + \overline{\mu _{j}})\Gamma (\lambda _{j}(a - 1 + it) \right. \\&\quad \left. + 1 - \mu _{j}) \sin (\pi (\lambda _{j}(1-a- it) + \mu _{j})) \right| . \end{aligned} \end{aligned}$$

We can easily show that

$$\begin{aligned} |\sin (\pi (\lambda _{j}(1-a- it) + \mu _{j}))| \le \exp (\pi |\lambda _{j}t - \mathrm {Im}(\mu _{j})|). \end{aligned}$$

On the other hand, using Stirling’s formula we can show that, for $x > 0$,

$$\begin{aligned} \log {|\Gamma (x + iy)|}&= \frac{1}{2}\left( x - \frac{1}{2}\right) \log (x^{2} + y^2) - y \arctan (y / x) - x\\&\quad +\, \frac{1}{2}\log {2\pi } + \varphi (x, y)\\ \nonumber&\le \frac{1}{2}\left( x - \frac{1}{2}\right) \log (x^{2} + y^2) - \frac{\pi }{2}|y| + \frac{1}{2}\log {2\pi } + \varphi (x, y), \end{aligned}$$

where the function $\varphi (x, y)$ satisfies the inequality (cf. Binet’s first formula)

$$\begin{aligned} |\varphi (x, y)| \le \Bigg |\int _{0}^{\infty }\left( \frac{1}{2} - \frac{1}{t} + \frac{1}{e^{t} - 1} \right) \frac{e^{-t(x+iy)}}{t}\mathrm{d}t \Bigg | \le \frac{1}{10x}. \end{aligned}$$

From these inequalities, we find that (note that ${\text {Re}}(\lambda _{j}(a - 1 + it) + 1 - \mu _{j}) \ge 0$ since $a > 1 + \mu _{M}/\lambda _{m}$)

$$\begin{aligned}&\left| \Gamma (\lambda _{j}(a + it) + \overline{\mu _{j}})\Gamma (\lambda _{j}(a - 1 + it) + 1 - \mu _{j}) \sin (\pi (\lambda _{j}(1-a- it) + \mu _{j})) \right| \\&\quad \quad \le 2\pi \exp \left( \frac{1}{5(\lambda _{j}(a - 1) - \mathrm {Re}(\mu _{j}))} \right) \\&\qquad \quad \times \,\left( (a\lambda _{M} + \mu _{M} + 1)^2 + (\lambda _{M}|t| + \mu _{M})^2 \right) ^{\frac{\lambda _{j}}{2}(2a - 1)}. \end{aligned}$$

Hence we have

$$\begin{aligned}&\frac{Q^{2a - 1}}{\pi ^{r}} \prod _{j = 1}^{r} \left| \Gamma (\lambda _{j}(a + it) + \overline{\mu _{j}})\Gamma (\lambda _{j}(a - 1 + it) \right. \\&\quad \left. + 1 - \mu _{j}) \sin (\pi (\lambda _{j}(1-a- it) + \mu _{j})) \right| \\&\quad \quad \le c_{{\mathcal {L}}}(a)Q^{2a - 1} \left( (a\lambda _{M} + \mu _{M} + 1)^2 + (\lambda _{M}|t| + \mu _{M})^2 \right) ^{\frac{d_{\mathcal {L}}}{4}(2a - 1)}, \end{aligned}$$

which completes the proof. $\square$

## Proof of Theorem

Now we are ready to prove our main theorem. We again put

$$\begin{aligned} \lambda _m{:}{=} \min _{1\le j \le r} \lambda _j,\quad \lambda _M{:}{=} \max _{1\le j \le r} \lambda _j,\quad \mu _M{:}{=} \max _{1\le j \le r} |\mu _j|, \end{aligned}$$

and let *a* be a real number satisfying $1 + \frac{\mu _{M} + 1}{\lambda _{m}}< a < \frac{1}{2} + \frac{n}{d_{\mathcal {L}}}$.

By Proposition 1 and Lemma 2, we have

$$\begin{aligned} |\gamma _n({\mathcal {L}})|&\le \frac{1}{2 \pi }\sum _{m = 1}^{\infty }\frac{|f(m)|}{m^{a}}\int _{-\infty }^{\infty } \frac{|G_{{\mathcal {L}}}(a + it)|}{\left( a^2 + t^2\right) ^{(n+1)/2}}\mathrm{d}t \\&\le \frac{c_{{\mathcal {L}}}(a)}{\pi }Q^{2a - 1}\sum _{m = 1}^{\infty }\frac{|f(m)|}{m^{a}} \int _{0}^{\infty }\\&\quad \times \frac{\left( (a\lambda _{M} + \mu _{M} + 1)^2 + (\lambda _{M}t + \mu _{M})^2 \right) ^{\frac{d_{\mathcal {L}}}{4}(2a - 1)}}{(a^2 + t^2)^{(n + 1)/2}}\mathrm{d}t, \end{aligned}$$

where

$$\begin{aligned} c_{{\mathcal {L}}}(a) = 2^{r}\exp \left( \frac{1}{5}\sum _{j = 1}^{r} \frac{1}{\lambda _{j}(a - 1) - \mathrm {Re}(\mu _{j})} \right) . \end{aligned}$$

We divide the region of integration into two as follows:

7 $$\begin{aligned} \left( \int _{0}^{A} + \int _{A}^{\infty } \right) \frac{\left( (a\lambda _{M} + \mu _{M} + 1)^2 + (\lambda _{M}t + \mu _{M})^2 \right) ^{\frac{d_{\mathcal {L}}}{4}(2a - 1)}}{(a^2 + t^2)^{(n + 1)/2}} \mathrm{d}t \,=:\, J_1 + J_2 \end{aligned}$$

with $A = a + \frac{1}{\lambda _{M}}$. We estimate $J_1$ and $J_2$ in the following manner:

$$\begin{aligned} J_1&\le 2^{\frac{3}{4}d_{\mathcal {L}}(2a - 1)}\int _{0}^{A}\frac{(a\lambda _{M}) ^{\frac{d_{\mathcal {L}}}{2}(2a - 1)}}{a^{n + 1}}\mathrm{d}t \le (8\lambda _{M}^2)^{\frac{d_{\mathcal {L}}}{4}(2a - 1)}\frac{A}{a}a^{-n + \frac{d_{\mathcal {L}}}{2}(2a - 1)}\\&\le 2(8\lambda _{M}^2)^{\frac{d_{\mathcal {L}}}{4}(2a - 1)}a^{-n + \frac{d_{\mathcal {L}}}{2}(2a - 1)}, \end{aligned}$$

and

$$\begin{aligned} J_2&\le 2^{\frac{3}{4}d_{\mathcal {L}}(2a - 1)}\int _{A}^{\infty } \frac{(\lambda _{M}t)^{\frac{d_{\mathcal {L}}}{2}(2a - 1)}}{t^{n + 1}}\mathrm{d}t \le (8\lambda _{M}^{2})^{\frac{d_{\mathcal {L}}}{4}(2a - 1)}\int _{A}^{\infty } \frac{\mathrm{d}t}{t^{n + 1 - d_{\mathcal {L}}(2a - 1)/2}} \\&\le \frac{(8\lambda _{M}^{2})^{\frac{d_{\mathcal {L}}}{4}(2a - 1)}}{n - d_{\mathcal {L}}(2a - 1)/2} a^{-n + \frac{d_{\mathcal {L}}}{2}(2a - 1)}. \end{aligned}$$

Substituting the above into (7), we obtain

$$\begin{aligned} |\gamma _n({\mathcal {L}})|&\le \frac{c_{{\mathcal {L}}}(a)}{\pi } Q^{2a - 1} \\&\quad \times \,\left( \sum _{m = 1}^{\infty }\frac{|f(m)|}{m^{a}}\right) \\&\quad \times \, (8\lambda _{M}^2)^{\frac{d_{\mathcal {L}}}{4}(2a - 1)}a^{-n + \frac{d_{\mathcal {L}}}{2}(2a - 1)} \left( 2 + \frac{1}{n - \frac{d_{\mathcal {L}}(2a - 1)}{2}} \right) . \end{aligned}$$

Therefore putting

$$\begin{aligned} C_{{\mathcal {L}}}(a)= & {} \frac{2^rQ^{2a - 1}}{\pi } \exp \\&\times \,\left( \frac{1}{5}\sum _{j = 1}^{r} \frac{1}{\lambda _{j}(a - 1) - \mathrm {Re}(\mu _{j})} \right) \left( \sum _{m = 1}^{\infty }\frac{|f(m)|}{m^{a}}\right) \\&\times \, (8\lambda _{M}^2a^2)^{\frac{d_{\mathcal {L}}}{4}(2a - 1)} \end{aligned}$$

for $1 + \frac{\mu _{M} + 1}{\lambda _{m}}< a < \frac{1}{2} + \frac{n}{d_{\mathcal {L}}}$, we obtain

$$\begin{aligned}&|\gamma _n({\mathcal {L}})| \le C_{{\mathcal {L}}}(a) a^{-n} \left( 2 + \frac{1}{n - \frac{d_{\mathcal {L}}(2a - 1)}{2}} \right) , \end{aligned}$$

which completes the proof of our Theorem.

## References

[1] Adell JA, Lekuona A (2017). Fast computation of the Stieltjes constants. Math. Comput..

[2] Adell JA (2011). Asymptotic estimates for Stieltjes constants: a probabilistic approach, Proc. R. Soc. Lond. Ser. A Math. Phys. Eng. Sci..

[3] Berndt BC (1972). On the Hurwitz zeta-function. Rocky Mt. J. Math..

[4] Bombieri Enrico, Lagarias Jeffrey C (1999). Complements to Li’s criterion for the Riemann hypothesis. J. Number Theory.

[5] Briggs, W.E.: Some constants associated with the Riemann zeta-function, Mich. Math. J. **3**, 117–121 (1955–1956)

[6] Coffey MW (2013). Hypergeometric summation representations of the Stieltjes constants. Analysis (Munich).

[7] Coffey MW (2014). Series representations for the Stieltjes constants. Rocky Mt. J. Math..

[8] Hashimoto Y, Iijima Y, Kurokawa N, Wakayama M (2004). Euler’s constants for the Selberg and the Dedekind zeta functions. Bull. Belg. Math. Soc..

[9] Ihara, Y.: On the Euler–Kronecker constants of global fields and primes with small norms. In: Ginzburg, V. (ed.) Algebraic Geometry and Number Theory. In Honor of Vladimir Drinfeld’s 50th Birthday, Progress in Mathematics, vol. 850, pp. 407–451. Birkhäuser Boston, Cambridge, MA (2006)

[10] Israilov MI (1981). The Laurent expansion of the Riemann zeta function (in Russian). Mat. Inst. Steklova.

[11] Knessl C, Coffey MW (2011). An effective asymptotic formula for the Stieltjes constants. Math. Comp..

[12] Matsuoka Y (1984). Euler, Generalized, constants associated with the Riemann zeta function, Number Theory and Combinatorics. Japan, : (Tokyo, Okayama and Kyoto, 1984).

[13] Mitrović D (1962). The signs of some constants associated with the Riemann zeta-function. Mich. Math. J.

[14] Moree P, Pintz J, Rassias M (2018). Irregular Behaviour of Class Numbers and Euler–Kronecker Constants of Cyclotomic Fields: The Log Log Log Devil at Play. Irregularities in the Distribution of Prime Numbers.

[15] Overholt M (2014). A Course in Analytic Number Theory, Graduate Studies in Mathematics.

[16] Saad Eddin S (2013). Explicit upper bounds for the Stieltjes constants,. J. Number Theory.

[17] Saad Eddin S (2017). Applications of the Laurent–Stieltjes constants for Dirichlet L-series. Proc. Jpn. Acad. Ser. A.

[18] Saad Eddin. S (2018). The signs of the Stieltjes constants associated with the Dedekind zeta-function. Proc. Jpn. Acad..

[19] Steuding J (2007). Value-Distribution of $$L$$-Functions.

[20] Tsfasman, M.A.: Asymptotic behaviour of the Euler–Kronecker constant. In: Ginzburg, V. (ed.) Algebraic Geometry and Number Theory. In Honor of Vladimir Drinfeld’s 50th Birthday, Progress in Mathematics, vol. 850, pp. 453–458. Birkhäuser Boston, Cambridge, MA (2006)

